# Saudi Oncology Society clinical management guidelines for prostate cancer

**DOI:** 10.4103/0974-7796.78550

**Published:** 2011-03

**Authors:** Ashraf J Abusamra, Shouki Bazarbashi, Yasser Bahader, Hussain Kushi, Dany Rabbah, Naser Al Bogami, Khalid Al Ghamdi, Abdullah Al Ghamdi, Khaled Balaraj, Raouf Seyam, Mohammed Al Otaibi, Eyad Al Saeed

**Affiliations:** Departments of Surgery and Oncology, King Abdul-Aziz Medical City, Jeddah, Saudi Arabian National Guard Health Affairs, Jeddah, Saudi Arabia

**Keywords:** Prostate, cancer, guidelines, Saudi, management

## Abstract

In this report, guidelines for the evaluation, medical and surgical management is presented. It is categorized according to the stage of the disease using the tumor node metastasis staging system, 7^th^ edition. The recommendations are presented with supporting level of evidence.

## INTRODUCTION

A total of 1869 cases of prostate cancer were recorded in Saudi Arabia between the year 1994 and 2004. The number of cases has been increasing steadily from 151 in 1994 to a peak of 243 cases in 2004.[1]

## 1. STAGING EVALUATION

Once diagnosis is confirmed, the following staging evaluation should be done:

**Table:** 

1.1.	CT or MRI abdomen and pelvis: this will only be done when the chance of lymph node metastasis is more than 10% based on Partin predictive tables (EL-2).[2–3] See Tables 1 and 2
1.2.	Bone scan: should be done if any of the following (EL-2):[4–5]
1.2.1.	PSA level more than 20
1.2.2.	Patients with bony pain
1.2.3.	Gleason score more than 7
1.2.4.	Patient with clinical stage T3 or 4

**Table 1:** Partin tables

Gleason score	Clinical stage T1c	Clinical stage T2a	Clinical stage T2b	Clinical stage T2c
Prediction of probability of organ-confined disease:				
Serum PSA = 0.0–2.5 ng/mL				
2–4	95 (89–99)	91 (79–98)	88 (73–97)	86 (71–97)
5–6	90 (88–93)	81 (77–85)	75 (69–81)	73 (63–81)
3 + 4 = 7	79 (74–85)	64 (56–71)	54 (46–63)	51 (38–63)
4 + 3 =7	71 (62–79)	53 (43–63)	43 (33–54)	39 (26–54)
8–10	66 (54–76)	47 (35–59)	37 (26–49)	34 (21–48)
Serum PSA = 2.6–4.0 ng/mL				
2–4	92 (82–98)	85 (69–96)	80 (61–95)	78 (58–94)
5–6	84 (81–86)	71 (66–75)	63 (57–69)	61 (50–70)
3 + 4 = 7	68 (62–74)	50 (43–57)	41 (33–48)	38 (27–50)
4 + 3 = 7	58 (48–67)	39 (30–48)	30 (22–39)	27 (18–40)
8–10	52 (41–63)	33 (24–44)	25 (17–34)	23 (14–34)
Serum PSA = 4.1–6.0 ng/mL				
2–4	90 (78–98)	81 (63–95)	75 (55–93)	73 (52–93)
5–6	80 (78–83)	66 (62–70)	57 (52–63)	55 (44–64)
3 + 4 = 7	63 (58–68)	44 (39–50)	35 (29–40)	31 (23–41)
4 + 3 = 7	52 (43–60)	33 (25–41)	25 (18–32)	21 (14–31)
8–10	46 (38–56)	28 (20–37)	21 (14–29)	18 (11–28)
Serum PSA 6.1–10 ng/mL				
2–4	87 (73–97)	76 (56–94)	69 (47–91)	67 (45–91)
5–6	75 (72–77)	58 (54–61)	49 (43–54)	46 (36–56)
3 + 4 = 7	54 (49–59)	35 (30–40)	26 (22–31)	24 (17–32)
4 + 3 = 7	43 (35–51)	25 (19–32)	19 (14–25)	16 (10–24)
8–10	37 (28–46)	21 (15–28)	15 (10–21)	13 (8–20)
Serum PSA > 10 ng/mL				
2–4	80 (61–95)	65 (43–89)	57 (35–86)	54 (32–85)
5–6	62 (58–64)	42 (38–46)	33 (28–38)	30 (21–38)
3 + 4 = 7	37 (32–42)	20 (17–24)	14 (11–17)	11 (7–17)
4 + 3 =7	27 (21–34)	14 (10–18)	9 (6–13)	7 (4–12)
8–10	22 (16–30)	11 (7–15)	7 (4–10)	6 (3–10)

**Table 2:** Partin’s table risk groups

	PSA		Gleason score		Clinical stage
Low risk	<10 ng/ml	AND	<6	AND	T1–T2a
Intermediate risk	10–20 ng/ml	OR	7	OR	T2b–T2c
High risk	>20 ng/ml	OR	8–10	OR	T3–T4

## 2. STAGING CLASSIFICATION

The TNM staging will be used[6]

### Primary tumor (T)

**Table:** 

TX	Tumor cannot be assessed
TO	No evidence of primary tumor
T1	Clinically not palpable or visible by imaging
T1a	Found incidental to other surgery; present in 5% or less of tissue
T1b	Found incidental to other surgery; present in 5% or more of tissue
T1c	Identified by needle biopsy
T2	Tumor confined within prostate
T2a	Involving half a lobe or less of prostate
T2b	Involving half a lobe
T2c	Involving both lobes
T3	Tumor extends through prostate capsule
T3a	Extends through one lobe
T3b	Extends through both lobes
T3c	Extends into seminal vesicles
T4	Involves structures other than seminal vesicles
T4a	Invades bladder neck, external sphincter, or rectum
T4b	Invades muscles and/or pelvic wall

### Regional lymph nodes (N)

**Table:** 

NX	Nodes cannot be assessed
NO	No regional node metastasis
N1	Single node metastasis, 2 centimeters (cm) or less at largest point
N2	Single node metastasis, 2 cm to 5 cm at largest point, or multiple nodes, no larger than 5 cm at largest point
N3	Metastasis larger than 5 cm in any node

### Distant metastasis (M)

**Table:** 

MX	Metastasis cannot be assessed
MO	No distant metastasis
M1	Distant metastasis
M1a	Distant lymph node(s) involved
M1b	Bone(s) involved
M1c	Other site(s) involved

## 3. MANAGEMENT OPTIONS

This will depend on the stage (localized vs metastatic), the risk group and life expectancy.[7]

**Table:** 

3.1.	Localized disease
3.1.1.	Low risk: options of therapy depend on the following factors:
3.1.1.1.	If the patient is asymptomatic with life expectancy less than 5 years: no further intervention required until he becomes symptomatic.[8–10] (EL-2)
3.1.1.2.	If asymptomatic with life expectancy between 5 and 10 years: active surveillance (involves active monitoring of the course of disease with the expectation to intervene if the cancer progresses).[8–10] (EL-2)
3.1.1.3.	If asymptomatic with life expectancy more than 10 years: options include active surveillance[10] or radical prostatectomy (RP)[11] or EBRT.[12] (EL-2)
3.1.1.4.	All RPs should be done in tertiary care centers by high-volume surgeons.[13] (EL-2)
3.1.1.5.	Lymphadenectomy is usually omitted if the chance of being positive is less than 2%.[14] (EL-2)
3.1.1.6.	3D conformal EBRT or Intensity-modulated EBRT (IMRT) are the only acceptable form of radiation therapy. The minimum dose should be not less than 72 Gy.[12,15–19] (EL-2)
3.1.1.7.	The choice of therapy should depend on the patient’s general condition, his preference and side-effect profile.
3.1.2.	Intermediate risk: options of therapy depend on the following factors:
3.1.2.1.	If life expectancy less than 5 years: patient will have no further intervention until he becomes symptomatic.[8–10] (EL-2)
3.1.2.2.	If life expectancy between 5 and 10 years: options include active surveillance[10] or EBRT+ 6 months of androgen deprivation therapy (ADT)[12,20] or RP.[11] (EL-2).
3.1.2.3.	If life expectancy more than 10 years: Options are RP with extended lymphadenectomy[11,21] (EL-1) or EBRT + 6 months of ADT (Radiation therapy to cover pelvic lymph nodes if chance of involvement is more than 15%).[12,15–16] (EL-2)
3.1.2.4.	Patients who have pT3 should undergo adjuvant EBRT to prostatic bed (66 Gy).[22–26] (EL-2)
3.1.2.5.	Patients who have positive margin following RP might undergo adjuvant EBRT to prostatic bed[22–26] or surveillance.[27–28] (EL-2)
3.1.3.	High risk: Options include RP + extended lymphadenectomy[29–30] (EL-3) or EBRT (to include pelvic lymph nodes) with 2–3 years of ADT.[31–37] (EL-1)
	Patients who have advanced local disease and are unfit for the above two options may be given ADT.[38] (EL-1)
3.1.4.	Follow up after curative therapy: Patients should have a disease-specific history, serum PSA and digital rectal examination (DRE) at 3, 6 and 12 months after therapy, then every 6 months for 3 years and then annually.[39] (EL-3)
3.1.5.	Management of recurrence post RP:
3.1.5.1.	Definition: recurrence post-radical prostatectomy is defined by PSA level more than 0.2 ng/dl.[40–41]
3.1.5.2.	Factors helping to differentiate local relapse or distant metastasis are: the timing of PSA increase after surgery, PSA doubling time (PSADT) and the stage and Gleason score at the time of surgery.[42,43]
3.1.5.3.	Management will depend on the time of detectable PSA level.[43–49] (EL-2);
3.1.5.3.1.	If PSA is increased in the initial two years of RP, this likely indicates systemic disease. However in order not to miss local recurrence, repeat PSA will be done after 3 months.
3.1.5.3.1.1.	If PSADT is less than 4 months then patient should be treated with ADT (see below).
3.1.5.3.1.2.	If PSADT more than 12 months this likely indicates local recurrence and patient should receive salvage EBRT.
3.1.5.3.1.3.	If PSADT is between 4 and 12 months then patient may receive combination of salvage EBRT and hormonal therapy.
3.1.5.3.2.	If PSA increase occurs after two years of surgery, then this likely indicates local recurrence. However in order not to miss systemic disease repeat PSA will be done after 3 months:
3.1.5.3.2.1.	If PSADT is less than 4 months then patient should be treated with hormone therapy (see below)
3.1.5.3.2.2.	If PSADT more than 12 months this likely indicates local recurrence and patient should receive salvage EBRT before PSA reaches 0.5 ng/dl
3.1.5.3.2.3.	If PSADT is between 4 and 12 months then patient may receive combination of salvage EBRT and hormonal therapy
3.1.5.3.3.	Bone scan and CT scan are of no diagnostic value unless PSA value is higher than 20 ng/dl[50–52] (EL-2).
3.1.6.	Management of local recurrence after EBRT
3.1.6.1.	Definition: a PSA rise 2 ng/mL above the PSA nadir is the most reliable indication for recurrence[53–54] (EL-2). However local recurrence is defined by the presence of all of the following:[55–56]
3.1.6.1.1.	A prostatic biopsy showing malignant cells 18 months or longer after EBRT
3.1.6.1.2.	Associated rise in PSA
3.1.6.1.3.	No evidence of distant metastasis documented by CT scan or MRI and bone scan
3.1.6.2.	Options of therapy include: observation up to PSA of 10 ng/dl, complete androgen blockade, and intermittent androgen deprivation.[57] In carefully selected patients, salvage prostatectomy, cryosurgery and brachytherapy might be considered.[58–61]
3.2.	Advanced disease (including recurrence and metastasis):
3.2.1.	Hormone responsive disease:
3.2.1.1.	ADT palliates symptoms and reduces the risk for potentially catastrophic sequelae of advanced disease (spinal cord compression, pathological fractures, ureteral obstruction, extraskeletal metastasis).[62–64] (EL-1)
3.2.1.2.	Options of therapy include: orchiectomy, LHRH agonists, anti-androgen monotherapy, and complete androgen blockade (CAB) continuous or intermittent.[62–64]
3.2.1.3.	High-risk patients with high PSADT, high initial PSA and symptomatic patient should preferably receive combined androgen blockade, while patients with low initial PSA, low PSADT and minimal symptoms may receive castration alone.[65–72] (EL-2)
3.2.1.4.	Castrate level of testosterone should be less than 20 ng/dl (0.7 nmol/L).[73–74]
3.2.1.5.	In case of intermittent androgen blockade (EL-2), the following should be observed:[75–89]
3.2.1.5.1.	Complete androgen blockade (anti-androgen and LHRH) should be used
3.2.1.5.2.	Initial induction cycle should be between 6 and 9 months
3.2.1.5.3.	Treatment is usually stopped only if the patient is compliant, showed PSA response with PSA less than 4 ng/dl in patients with metastatic disease and less than 0.5 ng/dl in recurrent disease post-local therapy
3.2.1.5.4.	Therapy is re-instituted for 3–6 months if PSA reaches 10–15 ng/dl in metastatic disease or 4 ng/dl in recurrent disease post-local therapy. Cycles repeated as above.
3.2.2.	Castrate resistant prostate cancer (CRPC):
3.2.2.1.	Definition: Two consecutive rises in PSA in the presence of castrate level of testosterone.
3.2.2.2.	Options of therapy include:
3.2.2.2.1.	In patients on monotherapy with LHRH agonist only or had orchiectomy as monotherapy: non-steroidal anti-androgen should be added.[90–91] (EL-2)
3.2.2.2.2.	In patients on combination therapy (complete androgen blockade: LHRH agonist (or orchiectomy) + non-steroidal anti-androgen), anti-androgen withdrawal should be done.[90–94] (EL-2)
3.2.2.2.3.	Patients who fails above measure can have one of the following options: anti-androgen substitution (bicalutamide or nilutamide)[95] (EL-2), Ketoconazole[94] (EL-2), systemic chemotherapy.
3.2.2.2.4.	Systemic chemotherapy in the form of Docetaxel and prednisone should be offered only to patients with performance status 0-2 by ECOG scale.[96–98] (EL-1)
3.2.2.2.5.	Patients with CRPC who were on LHRH agonists should continue on them indefinitely.[99–101] (EL-3)
3.2.2.2.6.	Patients who fail Docetaxel-based chemotherapy should be offered second-line chemotherapy with Cabazitaxel and prednisone if in good performance status.[102] (EL-1)
3.2.2.2.7.	Patients with advanced disease and bone metastatic should receive zoledronic acid[103–105] (EL-1) or Rank-ligand antibodies (Denosumab) therapy every 4 weeks.[106] (EL-1)
